# Non-Invasive Multimarker Strategy Combining IL-17A, Neutrophil–Albumin Ratio, and Fecal Calprotectin for Accurate Discrimination of IBD from IBS-D

**DOI:** 10.3390/ijms27125450

**Published:** 2026-06-16

**Authors:** Gamal Othman, Maysaa El Sayed Zaki, Nader Elmalki Elmalki, Abdelnaser A. Badawy, Samir A. Afifi

**Affiliations:** 1 Department of Basic Medical Sciences, College of Medicine, AlMaarefa University, Riyadh 24341, Saudi Arabia; 2 Clinical Pathology, Faculty of Medicine, Mansoura University, Mansoura 24341, Egypt; 3 Internal Medicine Department, Faculty of Medicine, Mansoura University, Mansoura 24341, Egypt; 4 Department of Biochemistry, Faculty of Medicine, Northern Border University, Arar City 35516, Saudi Arabia; 5 Internal Medicine Department, Faculty of Medicine, Zagazig University, Zagazig 41233, Egypt

**Keywords:** inflammatory bowel disease, IBS-D, IL-17A, neutrophil–albumin ratio, fecal calprotectin

## Abstract

Differentiating inflammatory bowel disease (IBD) from diarrhea-predominant irritable bowel syndrome (IBS-D) remains a major clinical challenge due to overlapping symptoms and the limited specificity of single biomarkers. A reliable, non-invasive multimarker approach is needed to improve diagnostic accuracy and reduce unnecessary endoscopic procedures. To evaluate the diagnostic performance of serum interleukin-17A (IL-17A), neutrophil-to-albumin ratio (NAR), and fecal calprotectin (FCP), individually and in combination, for discriminating IBD from IBS-D and healthy controls in Egyptian patients. In this case–control study, 300 participants (100 with IBD, 100 with IBS-D, and 100 healthy controls) were enrolled. Serum IL-17A, NAR, and FCP were measured, and subgroup analysis was performed for infected and non-infected IBS-D patients. Diagnostic performance was assessed using receiver operating characteristic (ROC) curve analysis, with optimal cutoffs determined by the Youden index. A combined biomarker model was constructed using logistic regression. All biomarkers demonstrated a significant stepwise increase from healthy controls to IBS-D and IBD (*p* < 0.001). IL-17A, NAR, and FCP were elevated in IBS-D compared with controls, indicating low-grade inflammation, but were highest in IBD. No significant differences were observed between infected and non-infected IBS-D patients. Among individual markers, NAR showed the highest diagnostic accuracy (AUC = 0.923), followed by FCP (AUC = 0.884) and IL-17A (AUC = 0.859). The combined model significantly improved performance (AUC = 0.973), achieving 89% sensitivity and 96% specificity. IBS-D is associated with measurable systemic and intestinal inflammation independent of infection status. The combined biomarker model integrating IL-17A, neutrophil–albumin ratio, and fecal calprotectin demonstrated promising discriminatory performance for differentiating IBD from IBS-D. These findings suggest the potential applicability of combined non-invasive biomarkers in future diagnostic stratification approaches. However, the model was developed and evaluated within a single cohort, and external validation in independent populations is required before future potential clinical application. A multimarker diagnostic panel integrating IL-17A, neutrophil–albumin ratio, and fecal calprotectin demonstrated promising diagnostic performance for differentiating inflammatory bowel disease from IBS-D. The combined model may contribute to future diagnostic stratification strategies in patients with chronic diarrhea. However, these findings were derived from a single cohort and require validation in independent populations before broader clinical application.

## 1. Introduction

Inflammatory bowel diseases (IBD), including ulcerative colitis (UC) and Crohn’s disease (CD), as well as functional gastrointestinal disorders such as irritable bowel syndrome (IBS), represent major causes of chronic gastrointestinal morbidity globally. The prevalence of IBD continues to rise in both Western countries and newly industrialized regions, significantly contributing to healthcare costs and a reduced quality of life for affected individuals [1]. Accurate and accessible biomarkers remain essential for diagnosing, classifying, and monitoring disease activity [2].

A hallmark of IBD is mucosal inflammation, characterized by epithelial disruption, neutrophil infiltration, and cytokine-driven immune activation [3]. Among these cytokines, interleukin-17A (IL-17A), produced predominantly by Th17 cells, plays a crucial role in host defense and the pathogenesis of chronic intestinal inflammation. Elevated IL-17A levels have been demonstrated in patients with active IBD and correlate with disease severity [4,5]. IL-17A promotes neutrophil recruitment by inducing the production of chemokines and inflammatory mediators, making it a potential diagnostic and therapeutic biomarker [6].

Neutrophils play a significant role in intestinal inflammation by inducing oxidative stress, promoting proteolytic activity, and forming neutrophil extracellular traps (NETs) [3]. As inflammation progresses, systemic markers such as serum albumin decline due to protein loss, malnutrition, and cytokine-mediated suppression of hepatic synthesis [7]. Based on these dynamics, composite indices integrating neutrophil count and albumin have shown promise. The Neutrophil-to-Albumin Ratio (NAR) has emerged as a novel inflammation-related biomarker and has recently been shown to correlate with symptom severity and cytokine levels in IBD patients [8].

Fecal calprotectin (FCP) remains one of the most widely used non-invasive markers for assessing intestinal inflammation. Calprotectin, a neutrophil-derived protein, is stable in stool and strongly correlates with mucosal inflammation. It reliably differentiates IBD from functional disorders and guides decisions regarding endoscopy [9].

Despite advances, few studies have concurrently examined IL-17A, NAR, and FCP within the same cohort to evaluate their relative diagnostic performance. Furthermore, the potential combined value of systemic inflammatory markers alongside fecal markers has not been sufficiently explored.

Differentiating inflammatory bowel disease (IBD), including Crohn’s disease and Ulcerative colitis, from diarrhea-predominant irritable bowel syndrome (IBS-D) using biomarkers is clinically essential because these conditions differ fundamentally in pathophysiology, prognosis, and management despite overlapping symptoms such as chronic diarrhea and abdominal pain. IBD is an immune-mediated inflammatory disorder requiring early anti-inflammatory or biologic therapy to prevent complications, whereas IBS-D is a functional disorder without structural inflammation, and misclassification may lead either to delayed treatment and disease progression in IBD or unnecessary invasive procedures and overtreatment in IBS-D. Non-invasive biomarkers such as fecal calprotectin and C-reactive protein provide objective evidence of intestinal inflammation, enabling discrimination between inflammatory and functional disease, reducing the need for colonoscopy, and improving diagnostic triage. Differentiating inflammatory bowel disease (IBD), including Crohn’s disease and ulcerative colitis, from diarrhea-predominant irritable bowel syndrome (IBS-D) remains clinically important because these conditions differ substantially in pathophysiology, prognosis, and management despite overlapping gastrointestinal symptoms. IBD is a chronic immune-mediated inflammatory disorder requiring early therapeutic intervention to prevent complications, whereas IBS-D is considered a functional gastrointestinal disorder without overt structural inflammation [10,11,12]. Non-invasive biomarkers such as fecal calprotectin and C-reactive protein have improved the ability to distinguish inflammatory from functional bowel disorders and may support more efficient diagnostic stratification [13,14]. In addition, biomarkers may assist in disease monitoring and therapeutic decision-making in IBD [14,15].

To our knowledge, relatively few studies have concurrently evaluated IL-17A, neutrophil–albumin ratio (NAR), and fecal calprotectin within the same cohort of IBS-D and IBD patients. Multimarker approaches may provide better diagnostic discrimination than individual biomarkers alone; however, further validation in independent cohorts is required before routine clinical application can be recommended.

This study aimed to: Compare demographic, clinical, and laboratory characteristics among IBS-D, IBD, and healthy control groups. Evaluate differences in IL-17A, NAR, and FCP between groups. Assess the diagnostic accuracy of IL-17A, NAR, FCP, and a combined IL-17A + NAR model using ROC curve analysis.

## 2. Result

### 2.1. Demographic and Clinical Characteristics

**Figure 1:**
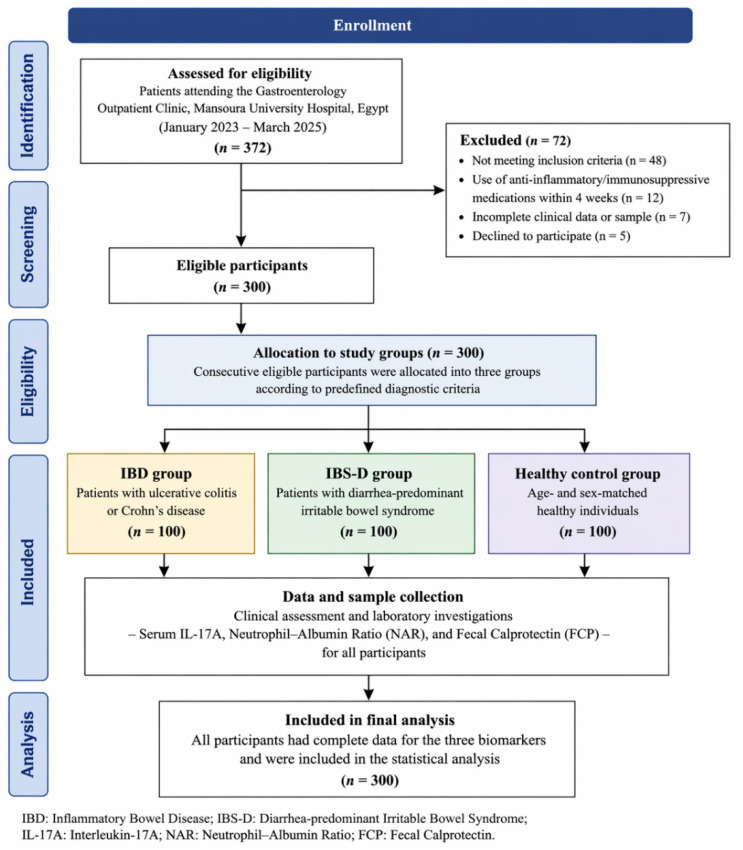
Study flow diagram showing participant selection, exclusion criteria, and allocation into study groups.

A total of 300 participants were included, equally divided among the IBS-D (*n* = 100), IBD (*n* = 100), and health control (*n* = 100) groups. Age differed significantly between groups (*p* = 0.0288). Post-infectious rates were highest in the IBS-D group (54%), moderate in the IBD group (47%), and absent in healthy controls (0%), demonstrating a strong association across groups (*p* < 0.001). Sex distribution did not differ significantly across groups (*p* = 0.078). IBS symptom severity (IBS-SSS) was significantly elevated in the IBS-D group (262.0 ± 51.86) compared with IBD and healthy subjects, who both scored 0 (*p* < 0.001), Table 1, Figure 1.

Exploratory subgroup analysis comparing ulcerative colitis (UC) and Crohn’s disease (CD) patients demonstrated no statistically significant differences in serum IL-17A, NAR, or fecal calprotectin levels between the two IBD subtypes (*p* > 0.05 for all comparisons). Although biomarker levels were numerically slightly higher in UC patients, the overall inflammatory profiles remained comparable between UC and CD. These findings suggest that the evaluated biomarkers reflect common inflammatory mechanisms across IBD subtypes and support the use of pooled IBD analysis for the primary objective of discriminating IBD from IBS-D.

IBS-SSS was assessed only in participants diagnosed with IBS-D according to Rome IV criteria. IBS-SSS was assessed only in the IBS-D group because the scoring system is not validated for inflammatory bowel disease or healthy control populations; therefore, values for these groups are presented as not applicable (N/A).

### 2.2. Comparison of Laboratory Markers Among IBS-D, IBD, and Healthy Controls

The mean serum IL-17A levels were significantly higher in IBD patients (7.38 ± 1.12 pg/mL) compared to IBS-D patients (6.20 ± 1.05 pg/mL) and healthy controls (5.01 ± 1.03 pg/mL). Similarly, the neutrophil–albumin ratio (NAR) increased progressively across groups, with mean values of 2.56 ± 0.46 in IBD, 1.84 ± 0.45 in IBS-D, and 1.39 ± 0.45 in healthy controls. Fecal calprotectin levels were also markedly elevated in IBD patients (78.47 ± 15.15 µg/g) compared to IBS-D (58.71 ± 16.08 µg/g) and healthy individuals (46.22 ± 13.34 µg/g). One-way ANOVA demonstrated highly significant differences among the three groups for all markers (*p* < 0.001). Post hoc analysis using Tukey’s test confirmed that all pairwise comparisons were statistically significant (*p* < 0.001), indicating a consistent increase from healthy controls to IBS-D and IBD patients (Table 2).

### 2.3. Laboratory Comparison Between Infected and Non-Infected IBS-D Patients

The comparison of laboratory parameters between infected and non-infected IBS-D patients revealed a consistent pattern of elevated inflammatory markers in IBS-D patients compared to healthy controls, regardless of infection status.

Specifically, serum IL-17A, NAR, fecal calprotectin, neutrophil count, and total leukocyte count were all significantly higher in both infected and non-infected IBS-D patients compared to healthy controls (P2 and P3 < 0.001 for all markers), confirming the presence of a systemic and intestinal inflammatory response in IBS-D.

The absence of statistically significant differences between clinically defined post-infectious and non-post-infectious IBS-D groups in the present cohort suggests that inflammatory biomarker levels may overlap between these subgroups. However, infection status was determined based on clinical history without microbiological confirmation or pathogen-specific testing. Therefore, the current findings should not be interpreted as excluding potential pathogen-specific inflammatory effects in IBS-D.

Additionally, serum albumin levels showed no significant differences across all groups (*p* = 0.979), indicating that albumin may not be a sensitive marker for detecting inflammatory differences in this cohort.

The NAR was calculated according to the following equation:

NAR = Absolute Neutrophil Count (×10^9^/L)/Serum Albumin (g/L)

Because serum albumin values showed minimal variation across study groups, whereas neutrophil counts differed substantially, the observed diagnostic performance of NAR in the present cohort likely reflects the stronger contribution of neutrophil elevation to the ratio.

Overall interpretation: These findings indicate that IBS-D is associated with a significant inflammatory profile compared with healthy individuals. Detailed laboratory comparisons are presented in Table 3.

### 2.4. ROC Performance of Individual and Combined Biomarkers for Discrimination of IBD

**Figure 2:**
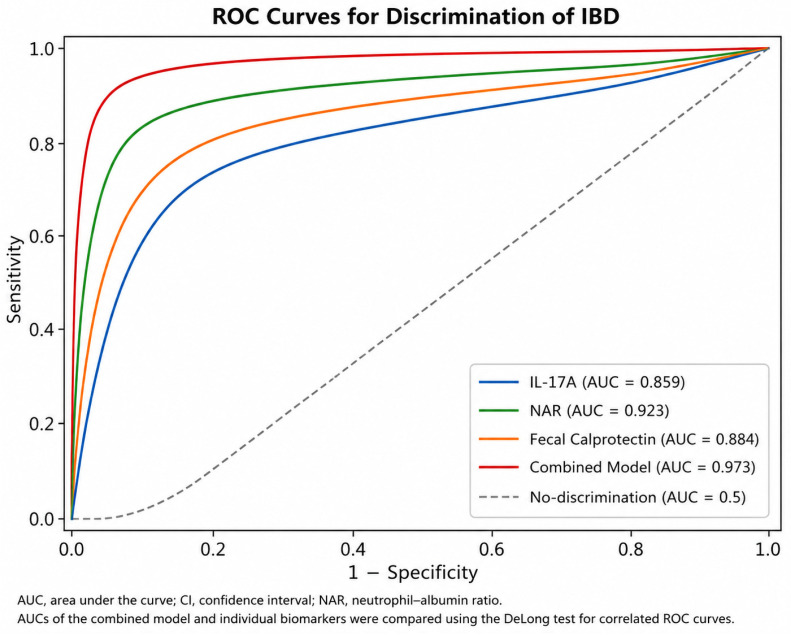
ROC performance of individual and combined biomarkers for discrimination of IBD.

ROC curve analysis demonstrated that NAR showed the highest diagnostic performance among the individual biomarkers for discriminating IBD from IBS-D (AUC = 0.923), followed by fecal calprotectin (AUC = 0.884) and IL-17A (AUC = 0.859). The revised ROC figure (Figure 2) was regenerated directly from the final statistical output and is fully consistent with the AUC values presented in Table 4, including the superior diagnostic performance of NAR among the individual biomarkers. Comparison of ROC curves using the DeLong test showed that the combined biomarker model had a significantly higher AUC than each individual biomarker alone (all *p* < 0.05), indicating superior diagnostic discrimination in distinguishing IBD from IBS-D.

### 2.5. Logistic Regression Model for the Combined Biomarker Panel

A binary logistic regression model was constructed to evaluate the combined diagnostic value of IL-17A, NAR, and fecal calprotectin for discriminating IBD from IBS-D. All three biomarkers were independently associated with IBD diagnosis.

The final regression equation was:

Logit(*P*) = −12.868 + (1.083 × IL-17A) + (1.893 × NAR) + (0.069 × Fecal Calprotectin)

where *P* represents the predicted probability of IBD.

IL-17A demonstrated an odds ratio (OR) of 2.95 (95% CI: 1.98–4.41, *p* < 0.001), NAR showed an OR of 6.64 (95% CI: 2.75–16.03, *p* < 0.001), and fecal calprotectin demonstrated an OR of 1.07 (95% CI: 1.04–1.10, *p* < 0.001). These findings indicate that increasing levels of each biomarker were independently associated with a higher probability of IBD.

IL-17A was also a significant independent predictor of IBD (OR = 2.95, 95% CI: 1.98–4.41, *p* < 0.001), suggesting that higher IL-17A levels were associated with nearly threefold higher odds of IBD. Table 5.

Fecal calprotectin demonstrated a smaller but statistically significant effect (OR = 1.07, 95% CI: 1.04–1.10, *p* < 0.001), indicating that higher calprotectin concentrations were independently associated with a greater probability of IBD.

All biomarkers remained statistically significant in the combined model, supporting their complementary diagnostic contribution. The negative intercept (−12.868) indicates that, in the absence of biomarker elevation, the baseline probability of IBD is low.

The final logistic regression equation was:

Logit(*P*) = −12.868 + (1.083 × IL-17A) + (1.893 × NAR) + (0.069 × Fecal Calprotectin)

where *P* represents the predicted probability of IBD. Higher calculated values indicate a greater likelihood of IBD than IBS-D.

For the combined logistic regression model, the reported cutoff value (0.49) corresponds to the predicted probability threshold that yielded the optimal balance between sensitivity and specificity in the ROC analysis. Predicted probabilities greater than 0.49 were classified as indicative of IBD.

## 3. Discussion

The present study evaluated the diagnostic utility of serum IL-17A, neutrophil–albumin ratio (NAR), and fecal calprotectin, individually and in combination, for differentiating inflammatory bowel disease (IBD) from diarrhea-predominant irritable bowel syndrome (IBS-D). The principal findings were: (1) all three biomarkers demonstrated a significant stepwise increase from healthy controls to IBS-D and IBD patients; (2) NAR exhibited the highest diagnostic performance among the individual biomarkers; and (3) a combined biomarker model integrating IL-17A, NAR, and fecal calprotectin achieved excellent discriminatory performance for distinguishing IBD from IBS-D.

The observed elevation of IL-17A in IBD patients is consistent with the established role of the IL-23/Th17 pathway in intestinal inflammation. Previous studies have demonstrated increased IL-17A expression in intestinal mucosa and circulation of patients with active IBD, supporting its role in mucosal immune activation and disease progression [16,17]. The intermediate IL-17A levels observed in IBS-D patients may reflect low-grade immune activation that has been increasingly recognized in subsets of IBS patients [18]. However, the cross-sectional design of the present study does not permit conclusions regarding causality or underlying mechanisms.

Fecal calprotectin remains one of the most widely accepted non-invasive biomarkers of intestinal inflammation. In agreement with previous studies and current clinical guidelines, fecal calprotectin levels were significantly higher in IBD patients than in IBS-D patients and healthy controls [19,20,21]. The modest elevations observed in IBS-D patients may reflect subtle mucosal immune activation, particularly in diarrhea-predominant and post-infectious IBS phenotypes [22,23].

Among the individual biomarkers evaluated, NAR demonstrated the highest diagnostic accuracy for discriminating IBD from IBS-D (AUC = 0.923). This finding is consistent with increasing evidence supporting composite inflammatory indices as practical biomarkers that integrate systemic inflammatory activity and host physiological status [21]. Although serum albumin levels alone did not significantly differ among study groups, the elevated NAR values observed in IBD patients likely reflect the contribution of neutrophil-mediated inflammation to disease pathogenesis.

The most clinically relevant finding of the present study was the excellent performance of the combined biomarker model incorporating IL-17A, NAR, and fecal calprotectin. The combined model achieved an AUC of 0.973 with high sensitivity and specificity and significantly outperformed each individual biomarker according to the DeLong test analysis. These findings support the concept that combining biomarkers representing distinct inflammatory pathways may improve diagnostic discrimination beyond that achieved by single markers alone [24,25]. Because the principal diagnostic challenge in routine practice is distinguishing IBD from IBS-D rather than differentiating patients from healthy individuals, ROC analyses were specifically focused on this clinically relevant comparison.

The present study also demonstrated that IBS-D patients exhibited measurable inflammatory alterations compared with healthy controls, including elevated IL-17A, NAR, fecal calprotectin, neutrophil count, and leukocyte count. These findings support growing evidence that IBS-D may involve persistent low-grade immune activation rather than representing a purely functional disorder [26]. Interestingly, no significant differences were observed between infected and non-infected IBS-D patients. However, because classification of post-infectious IBS-D was based on clinical history without microbiological confirmation, pathogen-specific inflammatory effects cannot be excluded.

### Strengths and Limitations

This study has several strengths. It is among the few studies to simultaneously evaluate cytokine-based, systemic, and fecal inflammatory biomarkers within the same cohort while also assessing their combined diagnostic performance. The inclusion of both infected and non-infected IBS-D subgroups provided additional insight into the inflammatory characteristics of IBS-D.

Several limitations should also be acknowledged. First, the single-center design and relatively limited sample size may restrict generalizability. Second, the cross-sectional design precludes assessment of temporal relationships and disease progression. Third, microbiological confirmation of post-infectious status and detailed gut microbiota characterization were not performed. Fourth, formal stratification of IBD patients according to disease activity was not undertaken. Finally, the combined biomarker model was developed and evaluated within the same cohort without internal resampling or external validation. Consequently, the reported diagnostic performance may overestimate real-world accuracy because of potential overfitting associated with the case–control design. Future studies should incorporate internal validation methods, such as bootstrapping or cross-validation, together with independent external validation cohorts.

In conclusion, the present study demonstrates that IL-17A, NAR, and fecal calprotectin are significantly associated with inflammatory bowel disease and that a combined multimarker approach provides excellent discrimination between IBD and IBS-D. These findings support the potential role of non-invasive biomarker panels in improving diagnostic stratification and reducing unnecessary invasive investigations. However, prospective multicenter validation studies are required before routine clinical implementation can be recommended.

## 4. Materials and Methods

### 4.1. Study Design and Participants

This was a case–control study including adults aged 18–65 years. Three groups were enrolled: diarrhea-predominant irritable bowel syndrome (IBS-D), inflammatory bowel disease (IBD), and healthy controls. The subjects were recruited from Mansoura University Hospital, Egypt, from January 2023 to March 2025. The study was conducted in accordance with the ethical principles outlined in the Declaration of Helsinki and its subsequent amendments. The study was approved by the Mansoura Faculty of Medicine’s ethical committee (R.26.01.3533), and enrolled subjects provided written informed consent. All participants were informed about the purpose of the study, the procedures involved, potential risks and benefits, and their right to withdraw at any time without affecting their medical care. Confidentiality and anonymity of all personal data were strictly maintained throughout the study, and data were used exclusively for scientific research purposes.

### 4.2. Reporting Guidelines

This study was conducted and reported in accordance with the Reporting of Studies Conducted using Observational Routinely Collected Health Data (RECORD) statement, an extension of the STROBE guidelines.

IBS-D was diagnosed using Rome IV criteria, with diarrhea ≥25% and constipation <25% of bowel movements [9]. Post-infectious IBS was defined according to established consensus criteria: acute infectious gastroenteritis followed by persistent IBS symptoms for ≥6 months [10]. IBD patients (Crohn’s disease or ulcerative colitis) were included as a disease control group, with diagnosis confirmed by endoscopy, histology, and cross-sectional imaging. In the present study, patients classified as “infected IBS-D” had a prior history of infectious gastroenteritis, consistent with post-infectious IBS-D rather than active infection at the time of sampling. Classification was based on structured clinical history, including preceding episodes of fever, vomiting, or ≥3 watery stools/day followed by persistent IBS-D symptoms. Participants with evidence of active gastrointestinal infection during enrollment were excluded. Routine microbiological stool cultures or serological testing were not systematically performed because the study focused on the clinical post-infectious IBS-D phenotype rather than acute infectious enteritis.

Health controls were asymptomatic individuals with no history of gastrointestinal disease. Exclusion criteria for all groups include major comorbidities, recent antibiotic or steroid use, pregnancy, or inability to complete study procedures.

IBD patients (Crohn’s disease or ulcerative colitis) were included based on established clinical, endoscopic, histopathological, and radiological criteria. Disease activity was assessed clinically and supported by standard inflammatory markers, including CRP and fecal calprotectin. Patients with severe acute flare requiring hospitalization or biologic induction therapy were not specifically targeted.

To minimize confounding effects on inflammatory biomarkers, detailed medication history was obtained for all participants. Individuals who had received systemic corticosteroids, immunosuppressive therapy initiation, biologic induction therapy, antibiotics, probiotics, or nonsteroidal anti-inflammatory drugs within four weeks before enrollment were excluded whenever applicable.

Participants with conditions known to affect inflammatory biomarkers were also excluded, including active infections, autoimmune diseases, chronic liver disease, chronic kidney disease, malignancy, metabolic or severe nutritional disorders, hematological diseases, and other systemic inflammatory conditions. Pregnant women and individuals unable to complete study procedures were also excluded.

Nutritional status was evaluated clinically and through serum albumin measurements; individuals with severe malnutrition or conditions significantly affecting albumin metabolism were excluded to reduce potential bias in NAR interpretation.

### 4.3. Sample Size

The health outcome for sample size estimation was the difference in inflammatory biomarkers (IL-17A, NAR, and fecal calprotectin) across the three independent groups (IBS-D, IBD, and healthy controls). Sample size was calculated using a one-way ANOVA framework with equal group allocation.

Because high-quality prior data comparing IL-17A and NAR across these groups were not available at the time of design, the study adopted a conservative, clinically meaningful effect size (Cohen’s f = 0.40), which is large according to conventional criteria. Using α = 0.05, power = 80%, and three equal groups, the required sample size would be *n* = 64 per group for a large effect size (f = 0.40).

To ensure robust power even under more modest effects (f = 0.30) and to accommodate potential exclusions, missing data, or non-normal biomarker distributions, the sample size was substantially increased.

Thus, recruiting 100 participants per group (total *n* = 300) provides:

>90% power to detect a large effect size
>80% power to detect a moderate-to-large effect size (f = 0.30–0.35)

Adequate precision for ROC curve analysis, where ≥100 diseased and ≥100 controls provide stable AUC estimation and narrow confidence intervals.

Sufficient sample size for subgroup analysis (e.g., post-infectious vs. non-post-infectious IBS-D).

Therefore, a final sample size of 100 participants per group was deemed appropriate and statistically justified for detecting clinically relevant differences in inflammatory biomarkers and ensuring stable ROC performance estimates.

Because prior studies had not simultaneously evaluated IL-17A, NAR, and fecal calprotectin in IBS-D, IBD, and healthy controls, precise effect size estimates were unavailable at the time of study design. Therefore, sample size estimation was based on a clinically meaningful large effect size (Cohen’s f = 0.40), consistent with Cohen’s conventional classification for ANOVA models, where f values of 0.10, 0.25, and 0.40 represent small, medium, and large effects, respectively. This assumption was considered reasonable given previously reported substantial differences in inflammatory biomarkers between inflammatory and functional gastrointestinal disorders.

### 4.4. Clinical Assessment

All participants underwent structured clinical evaluations, including demographics, symptom duration, bowel habit subtyping, and medication use. IBS symptom severity was assessed in the IBS-D group using the validated IBS Severity Scoring System (IBS-SSS) [27]. Post-infectious phenotype was identified via structured interview regarding preceding infectious gastroenteritis (fever, vomiting, or ≥3 watery stools/day) [28]. The IBS Severity Scoring System (IBS-SSS) was evaluated only in IBS-D patients because the instrument is specifically designed and validated for IBS assessment and does not apply to IBD patients or healthy controls.

### 4.5. Blood Sampling and Laboratory Measurements

Fasting blood samples were collected in the morning and processed within two hours. Serum IL-17A concentrations were measured using a commercially available enzyme-linked immunosorbent assay (ELISA), Human IL-17 Quantikine HS ELISA Kit (R&D Systems, Inc., Minneapolis, MN, USA), following manufacturer instructions.

Fecal Calprotectin was determined by a commercially available ELISA Kit (MyBioSource, Inc., San Diego, CA, USA).

Complete blood count, neutrophil count, and serum albumin were analyzed using automated hematology (CBC Analyzer, Sweal, Stockholm, Sweden) and biochemistry analyzers (Adaltis system, Adultism S.r.l. Headquarters, Production and Development Plant, Guidonia Montecelio, Italy).

The neutrophil-to-albumin ratio (NAR) was calculated by dividing the absolute neutrophil count (×10^9^/L) by serum albumin (g/L), in accordance with prior inflammatory biomarker studies [29]. C-reactive protein (CRP) and erythrocyte sedimentation rate (ESR) were included as standard inflammatory markers.

### 4.6. Statistical Analysis

Data distribution was assessed using the Shapiro–Wilk test and density plots. The Shapiro–Wilk test demonstrated approximate normal distribution for IL-17A (W = 0.982, *p* = 0.071), neutrophil–albumin ratio (NAR) (W = 0.978, *p* = 0.054), and fecal calprotectin (W = 0.975, *p* = 0.061), supporting the use of parametric analyses for the primary comparisons.

Normally distributed variables were expressed as mean ± SD and compared using one-way ANOVA followed by Tukey’s honest significant difference (HSD) post hoc correction for multiple comparisons. Categorical variables were compared using the χ^2^ or Fisher’s exact test. Subgroup comparisons between post-infectious and non-post-infectious IBS-D were performed using Welch’s *t* test or the Mann–Whitney *U* test, as appropriate. Non-normally distributed data were presented as median (IQR) and analyzed using the Kruskal–Wallis test followed by Dunn–Bonferroni correction for multiple comparisons.

Diagnostic performance of IL-17A, neutrophil-to-albumin ratio (NAR), fecal calprotectin (FCP), and a combined IL-17A + NAR model was evaluated using ROC curve analysis, with AUCs and 95% confidence intervals calculated by the DeLong method. Optimal cut-offs were determined using the Youden index. A binary logistic regression model was applied for combined biomarker assessment. All tests were two-sided (*p* < 0.05), and analyses were conducted using Python (version 3.14) and SPSS25. Receiver operating characteristic (ROC) analyses were reported with corresponding 95% confidence intervals for AUC, sensitivity, and specificity estimates to provide measures of diagnostic precision.

Receiver operating characteristic curve analysis was performed to evaluate the diagnostic performance of individual biomarkers and the combined logistic regression model. Predicted probabilities generated from the binary logistic regression equation were used to construct the ROC curve for the combined model. Comparisons between AUCs were performed using the DeLong test for correlated ROC curves. A *p* value < 0.05 was considered statistically significant.

IL-17A and NAR demonstrated strong discriminatory capacity between diseased and healthy individuals, supporting their potential role as accessible triage biomarkers, particularly in resource-limited settings. FCP demonstrated excellent diagnostic accuracy in identifying patients who required further IBD evaluation. No significant systemic inflammatory differences were observed between post-infectious and non-post-infectious IBS-D, indicating limited utility of routine inflammatory markers for IBS subtyping.

The combined biomarker model was constructed using binary logistic regression with IL-17A, neutrophil–albumin ratio (NAR), and fecal calprotectin as independent variables. Regression coefficients (β), odds ratios (ORs), and 95% confidence intervals (CIs) are presented to facilitate interpretation and reproducibility of the model.

## 5. Conclusions

In conclusion, IL-17A, neutrophil–albumin ratio, and fecal calprotectin demonstrated significant associations with IBD compared with IBS-D and healthy controls. The combined biomarker model showed promising diagnostic performance for differentiating inflammatory bowel disease from IBS-D and may represent a preliminary non-invasive diagnostic approach requiring further validation. Nevertheless, these findings were generated from a single cohort and should therefore be considered exploratory until confirmed in independent external populations through larger multicenter studies.

Among the evaluated biomarkers, the neutrophil–albumin ratio showed the highest individual diagnostic performance, while the combined biomarker model integrating IL-17A, NAR, and fecal calprotectin achieved excellent accuracy in discriminating IBD from IBS-D. These findings highlight the added value of a multimarker approach that captures the complementary aspects of systemic and mucosal inflammation.

Interestingly, no significant differences were observed between clinically defined post-infectious and non-post-infectious IBS-D patients for the evaluated inflammatory markers. However, because subgroup classification relied primarily on clinical history rather than microbiological confirmation, these findings should be interpreted cautiously. The present study, therefore, does not exclude potential pathogen-specific or active infection-related effects on inflammatory responses in IBS-D.

Collectively, this study suggests that integrating cytokine-based, hematological, and fecal biomarkers may improve non-invasive discrimination between IBD and IBS-D. However, the combined model requires external validation and confirmation using internal resampling methods before clinical implementation, or it can be used as a triage tool in patients with suspected bowel disorders. Future large-scale, longitudinal studies incorporating microbiome profiling and endoscopic validation are warranted to refine these biomarkers further and facilitate their translation into routine clinical practice.

## Figures and Tables

**Table 1:** Demographic and clinical characteristics.

Variable	IBS-D (*n* = 100)	IBD (*n* = 100)	Healthy Controls (*n* = 100)	*p* Value
Age (years), mean ± SD	38.09 ± 13.50	43.06 ± 13.72	39.79 ± 12.75	0.0288
Sex (Female/Male), *n*	41/59	45/55	56/44	0.078
Post-infectious status, *n* (%)	54 (54%)	47 (47%)	0 (0%)	<0.001
IBS-SSS, mean ± SD	262.00 ± 51.86	N/A	N/A	-

Abbreviations: IBS-D, diarrhea-predominant irritable bowel syndrome; IBD, inflammatory bowel disease; IBS-SSS, Irritable Bowel Syndrome Severity Scoring System; SD, standard deviation. Data are presented as mean ± standard deviation (SD) or number (%), as appropriate. *p* values represent overall comparisons among all three groups. Continuous variables were analyzed using one-way ANOVA, and categorical variables were compared using the chi-square test. IBS-SSS was assessed only in the IBS-D group because the scoring system is not validated for IBD patients or healthy controls; therefore, values for these groups are presented as not applicable (N/A).

**Table 2:** Comparison of laboratory markers among IBS-D, IBD, and healthy controls with 95% confidence intervals.

Marker	Healthy (*n* = 100)	IBS-D (*n* = 100)	IBD (*n* = 100)	*p* Value
IL-17A (pg/mL)	5.01 ± 1.03 (95% CI: 4.81–5.21)	6.20 ± 1.05 (95% CI: 5.99–6.41)	7.38 ± 1.12 (95% CI: 7.16–7.60)	<0.001
NAR	1.39 ± 0.45 (95% CI: 1.30–1.48)	1.84 ± 0.45 (95% CI: 1.75–1.93)	2.56 ± 0.46 (95% CI: 2.47–2.65)	<0.001
Fecal Calprotectin (µg/g)	46.22 ± 13.34 (95% CI: 43.58–48.86)	58.71 ± 16.08 (95% CI: 55.52–61.90)	78.47 ± 15.15 (95% CI: 75.47–81.47)	<0.001

Data are presented as mean ± standard deviation (SD) with corresponding 95% confidence intervals (95% CI). Overall comparisons among the three groups were performed using one-way ANOVA.

**Table 3:** Laboratory comparison between infected and non-infected IBS-D patients.

Marker	Healthy (*n* = 100)	Non-Infected IBS-D (*n* = 56)	Infected IBS-D (*n* = 44)	*p* Value	*P*1	*P*2	*P*3
IL-17A (pg/mL)	5.01 ± 1.03	6.27 ± 0.97	6.12 ± 1.15	<0.001	0.747	<0.001	<0.001
NAR	1.39 ± 0.45	1.87 ± 0.44	1.79 ± 0.45	<0.001	0.655	<0.001	<0.001
Fecal Calprotectin (µg/g)	46.22 ± 13.34	58.91 ± 14.62	58.45 ± 17.94	<0.001	0.987	<0.001	<0.001
Neutrophils (×10^9^/L)	55.88 ± 19.19	75.45 ± 18.96	72.42 ± 19.49	<0.001	0.712	<0.001	<0.001
Total Leukocyte Count (×10^9^/L)	58.8 ± 19.25	78.38 ± 19.04	75.67 ± 19.48	<0.001	0.764	<0.001	<0.001
Albumin (g/L)	40.21 ± 2.9	40.18 ± 2.91	40.3 ± 2.98	0.979	0.979	0.986	0.998

Post hoc definitions. *p* value: overall comparison among all study groups using one-way ANOVA. *P*1: comparison between infected and non-infected IBS-D patients. *P*2: comparison between infected IBS-D patients and healthy controls. *P*3: comparison between non-infected IBS-D patients and healthy controls.

**Table 4:** ROC performance of individual and combined biomarkers for discrimination of IBD.

Biomarker	AUC (95% CI)	Cutoff (95% CI)	Sensitivity % (95% CI)	Specificity % (95% CI)
IL-17A (pg/mL)	0.859 (0.807–0.911)	6.32	79 (70.0–86.1)	80 (71.0–87.1)
NAR	0.923 (0.884–0.962)	2.15	84 (75.3–90.6)	90 (82.4–95.1)
Fecal Calprotectin (µg/g)	0.884 (0.836–0.932)	66.85	81 (72.0–88.0)	82 (73.1–89.0)
Combined (IL-17A + NAR + Calprotectin)	0.973 (0.952–0.994)	0.49	89 (81.2–94.4)	96 (90.1–98.9)

Abbreviations: AUC, area under the curve; CI, confidence interval; NAR, neutrophil–albumin ratio. AUCs of the combined model and individual biomarkers were compared using the DeLong test for correlated ROC curves. Data are presented with corresponding 95% confidence intervals (95% CI) for AUC, sensitivity, and specificity estimates. The cutoff value of 0.49 for the combined biomarker model represents the predicted probability threshold derived from the binary logistic regression equation and selected using the Youden index for optimal discrimination between IBD and IBS-D.

**Table 5:** Binary logistic regression analysis for the combined biomarker model predicting IBD.

Variable	β Coefficient	Standard Error	Odds Ratio (OR)	95% CI	*p* Value
IL-17A (pg/mL)	1.083	0.205	2.95	1.98–4.41	<0.001
NAR	1.893	0.450	6.64	2.75–16.03	<0.001
Fecal Calprotectin (µg/g)	0.069	0.015	1.07	1.04–1.10	<0.001
Constant	−12.868	1.788	—	—	<0.001

Logit(*P*) = −12.868 + (1.083 × IL-17A) + (1.893 × NAR) + (0.069 × Fecal Calprotectin). Abbreviations: OR, odds ratio; CI, confidence interval; NAR, neutrophil–albumin ratio.

## Data Availability

The raw data supporting the conclusions of this article will be made available by the authors on request.
